# The Evolution of Cell Culture Systems to Study Hepatitis B Virus Pathogenesis and Antiviral Susceptibility

**DOI:** 10.3390/v17081057

**Published:** 2025-07-29

**Authors:** Thabani Sibiya, Lunga Xaba, Lulama Mthethwa, Anil A. Chuturgoon, Nokukhanya Msomi

**Affiliations:** 1 Discipline of Virology, University of KwaZulu-Natal, School of Laboratory Medicine and Medical Sciences and National Health Laboratory Service, Durban 4013, South Africa; xabal@ukzn.ac.za (L.X.); ldcmthethwa@gmail.com (L.M.); 2 Discipline of Medical Biochemistry and Chemical Pathology, School of Laboratory Medicine and Medical Sciences, College of Health Sciences, University of KwaZulu-Natal, Howard College Campus, Durban 4013, South Africa; chutur@ukzn.ac.za

**Keywords:** cell culture systems, HepG2-NTCP cells, hepatitis B virus (HBV)

## Abstract

The global burden of hepatitis B virus (HBV) remains high, with ongoing concerted efforts to eliminate viral hepatitis as a public health concern by 2030. The absence of curative treatment against HBV makes it an active area of research to further study HBV pathogenesis. In vitro cell culture systems are essential in exploration of molecular mechanisms for HBV propagation and the development of therapeutic targets for antiviral agents. The lack of an efficient cell culture system is one of the challenges limiting the development and study of novel antiviral strategies for HBV infection. However, the evolution of cell culture systems to study HBV pathogenesis and treatment susceptibility in vitro has made a significant contribution to public health. The currently available cell culture systems to grow HBV have their advantages and limitations, requiring further optimization. The discovery of sodium taurocholate co-transporting polypeptide (NTCP) as a receptor for HBV was a major breakthrough for the development of a robust cell model, allowing the study of de novo HBV infection through NTCP expression in the HepG2 hepatoma cell line. This review is aimed at highlighting the evolution of cell culture systems to study HBV pathogenesis and in vitro treatment susceptibility.

## 1. Introduction

The World Health Organization (WHO), in its 2024 report, estimated that 254 million people live with chronic HBV infection globally [1]. The U.S. Centers for Disease Control and Prevention (CDC) also reported that over 6 million children under the age of 5 years live with chronic HBV infection [2]. Moreover, the CDC estimated that HBV contributes to 820,000 deaths annually [2]. According to a WHO report, approximately 1.1 million HBV-related deaths occurred in 2022 [1], with over 60 million sub-Saharan Africans living with chronic HBV, of which 2.7 million are from South Africa in 2022 [1,3].

The availability of antiretroviral therapy (ART) and the HBV vaccine has tremendously changed the incidence of HBV [4,5]. Nucleos(t)ide analogs (NAs), such as tenofovir (TDF and TAF), lamivudine (3TC), and emtricitabine (FTC), are used to suppress HBV replication and are often combined in regimens for HIV/HBV co-infected patients. Entecavir is an alternative treatment when tenofovir is contraindicated [6]. Guidelines from the WHO, American Association for the Study of Liver Diseases (AASLD), European Association for the Study of the Liver (EASL), and Asian Pacific Association for the Study of the Liver (APASL) recommend first-line treatment with TDF, TAF, or entecavir to prevent liver disease progression and complications, such as cirrhosis and hepatocellular carcinoma (HCC) [1,7,8,9,10,11]. Treatment initiation is generally recommended for patients with active liver disease, significant fibrosis, or cirrhosis, and for specific populations, such as pregnant women and those co-infected with HCV, HIV, or HDV [7,9,12,13,14]. Despite effective viral suppression, long-term therapy is challenged by drug toxicity, resistance, and metabolic side effects [15].

The WHO has set an ambitious target to eliminate viral hepatitis by 2030; however, progress is limited by the lack of robust in vitro models for HBV research [16,17,18]. The discovery of sodium taurocholate co-transporting polypeptide (NTCP) as an HBV receptor enabled the development of the HepG2-NTCP cell line, which supports de novo HBV infection and facilitates studies on viral pathogenesis, antiviral screening, and drug toxicity [19,20,21,22]. This review examines the progress in developing cell culture systems to study HBV and highlights the HepG2-NTCP cell culture system as an emerging and promising approach. The HepG2-NTCP model shows promise for advancing research on the complete HBV life cycle, existing therapeutic strategies, and their mechanisms of action, potentially leading to a deeper understanding of HBV infection and more effective treatment.

## 2. HBV Biological Organization

HBV, a virus of the Hepadnaviridae family, primarily infects humans and can also infect chimpanzees, chacma baboons, and tree shrews [23,24]. It has up to ten genotypes and a partially double-stranded relaxed circular deoxyribonucleic acid (rcDNA) genome [23]. The virus exists in three forms: infectious Dane particles and non-infectious spherical and filamentous particles [23,24].

### 2.1. Organization and Structure of the Genome

**Figure 1:**
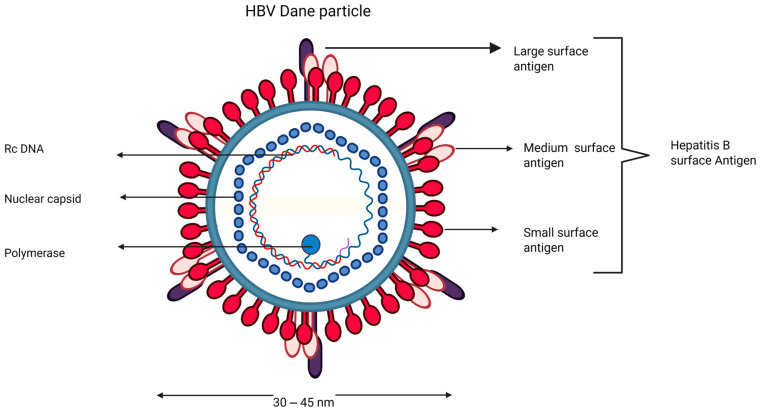
Structural representation of infectious HBV virions. HBsAg S, M, and L surface proteins on the lipid envelope. The lipid envelope surrounds the nucleocapsid (containing a relaxed circular DNA (rcDNA) and the viral DNA polymerase. Created in BioRender. Xaba, L. (2025) https://BioRender.com/i0t9boo (accessed on 18 July 2025).

The Dane particle of HBV is 30–42 nm in diameter (Figure 1) [24]. The HBV genome has four overlapping open reading frames (ORFs): S (surface proteins), C (core proteins), P (polymerase), and X (HBx protein). The multifunctional viral protein known as HBV X protein (HBx) controls a number of signaling pathways, activates transcription, advances the cell cycle, and is involved in deoxyribonucleic acid (DNA) repair and the breakdown of proteins [15]. The P ORF encodes a polymerase with reverse transcriptase and RNase H activity, which is required for viral replication [25,26]. HBV has a durable viral genome embedded in episomes and covalently closed circular deoxyribonucleic acid (cccDNA), which plays a significant role in the nuclei of infected hepatocytes. Understanding the biological organization of HBV and therapeutic drug targets is crucial for developing a cure. The inner nucleocapsid is icosahedral and contains the core antigen (HBcAg) enclosed by lipid-implanted surface proteins (HBsAg) [27] (Figure 1). Pre-S1 and pre-S2 are crucial parts of the virion protein envelope, involved in assembly, infection, replication, and host immune feedback [28,29,30]. Mutations in the pre-S gene can result in immunological evasion, liver dysfunction [31], and suppression of M protein expression [32]. HBV core gene expression produces two distinct homodimer proteins with distinct physiological properties: the core antigen (HBcAg) and the e antigen (HBeAg) [33]. The secreted form of HBcAg in the bloodstream is HBeAg, a marker of active viral replication of HBV [34,35,36].

Significant biomarkers of HBV include blood HBV DNA, anti-HBe antibody, hepatitis B e antigen (HBeAg), and hepatitis B core antigen (HBcAg) [37,38]. Infections caused by the virus are categorized as HBeAg-positive or HBeAg-negative based on the secreted form of HBcAg, HBeAg. Patients with HBeAg-positive infections have higher viral levels and robust disease progression [39]. In accordance with recommendations from major hepatology societies, key biomarkers such as HBeAg, HBsAg quantification, HBV DNA levels, and alanine aminotransferase (ALT) levels are used to guide therapy decisions [9]. Despite the availability of a safe and effective vaccine, no known treatment plan consistently eliminates chronic HBV.

### 2.2. The Viral Transmission of HBV and Replication Cycle

HBV is transmitted through infected bodily fluids [37], with the main route being during the perinatal period and early childhood [38,39]. Pregnant women can vertically transmit the virus to their offspring, leading to high levels of viremia and HBeAg positivity [38,39]. HBV replicates in hepatocytes [40], where HBsAg, particularly through the pre-S1 region, is crucial for viral interaction [41,42]. The virus enters hepatocytes via NTCP, a transporter [20], and nucleocapsid complexes are released from endocytic vesicles, generating transcription templates in the nucleus [43,44,45].

### 2.3. HBV Replication and the Contribution of Cell Division to HBV Resolution

The formation of cccDNA is a crucial step in the HBV life cycle, allowing the virus to establish a persistent infection in hepatocytes. After entering the nucleus, the capsid breaks down, releasing partially double-stranded relaxed circular DNA (rcDNA) [46]. This process involves DNA repair machinery and cellular enzymes, such as DNA polymerase κ and tyrosyl-DNA-phosphodiesterase 2 (TDP2) [47]. HBV replication within infected cells relies on cccDNA, which serves as a template for the transcription of pregenomic RNA (pgRNA) [48,49,50]. HBV polymerase interacts with host factors, such as heat shock protein 90, facilitating viral replication and allowing the terminal protein to engage with epsilon (ε) RNA [51,52,53].

**Figure 2:**
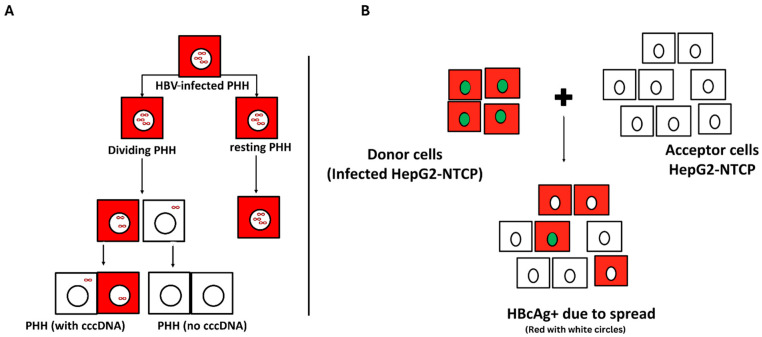
Schematic illustration of HBV covalently closed circular DNA (cccDNA) fate in HBV-infected PHH and HBV spread in HepG2-NTCP cells [57,58]. (**A**) Proliferation of HBV-infected hepatocytes results in the loss of cccDNA; this shows how individual cccDNA molecules are distributed during cell division [58]. Red-shaded squares represent cells infected with active HBV replication and cccDNA is represented by the infinity symbol (∞). (**B**) Schematic representation of experimental designs showing fate of HBV in two co-culture systems of HBV donor (red squared with green circles) and acceptor cells (white squares with white circles) in the presence of PEG [57]. Red squares with white circles are infected acceptor cells due to spread. PHH: primary human hepatocyte; NTCP: sodium taurocholate co-transporting polypeptide (adapted with permission from Professor Maura Dandri).

Cell division is crucial in determining the fate of cccDNA and the resolution of HBV infection [54]. Hepatocyte proliferation contributes to the reduction of cccDNA, which may be lost or unevenly distributed during mitosis (Figure 2A) [55,56]. Targeting HBV-infected cells and stimulating cell division may offer a potential therapeutic avenue. Some studies have developed a HepG2-NTCP cell culture system for efficient HBV production and spread; however, questions remain. A study by Michailidis, Pabon [57] found that enhanced HBV infection in HepG2-NTCP cells is NTCP-dependent (Figure 2B) [57].

## 3. Cell Culture Systems to Study HBV Pathogenesis

HBV cell culture systems generally lack the ability to propagate HBV [48]. Earlier studies described a method of generating full-length HBV genomes cable of being circularized in a manner similar to that of cccDNA, allowing for HBV replication when transfected into the Huh7 cell line [59]. These greater-than-genome-length HBV DNA constructs facilitate the production of viral RNA and their subsequent proteins in culture. Two well-defined human hepatoma cell lines, HepG2 and Huh7, support viral replication following transfection with an appropriate vector [48,60,61]. These transiently transfected cell lines offer a robust system useful in HBV replication and inhibition analysis, as seen with the extensive use of HepG22.2.15 in antiviral research [60].

### 3.1. Primary Human Hepatocytes

**Figure 3:**
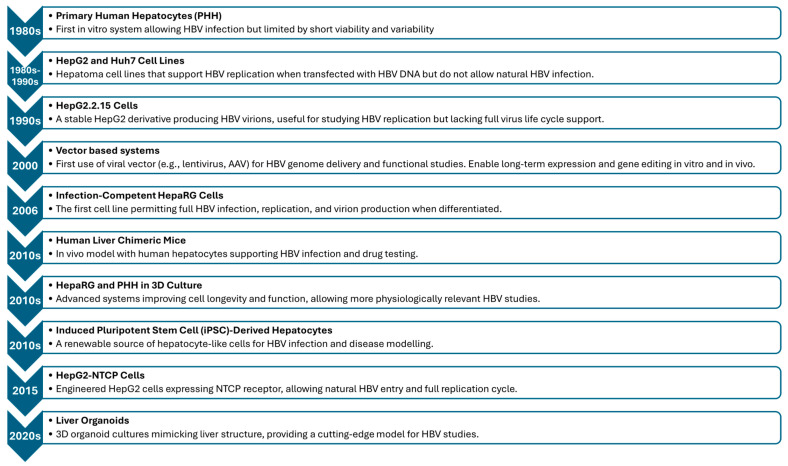
HBV cell culture systems in chronological order of development.

Primary human hepatocytes (PHHs) are used to assess hepatic metabolism, drug interactions, and toxicity in vitro [62,63]. They support the HBV life cycle (Figure 3), maintain liver function, and express host-specific factors [64,65,66,67]. However, their use is limited by factors such as limited donor availability [68], loss of differentiation [62], culture conditions influencing the longevity of cell lines, variability in infection susceptibility [69], and rapid dedifferentiation [62].

Recent advances in HBV research have included a combination of animal models (in vivo) and cell culture-based models using mouse models grafted with partially humanized livers to improve the efficiency of studying HBV replication. These in vivo and in vitro models for HBV research have been reviewed elsewhere [70].

PHHs are the preferred model for liver disease research because of their resemblance to the in vivo hepatic environment. High-activity PHHs have been harvested from liver tissue obtained during surgeries, offering improved selection methods for tissue sources and infection systems [71]. Cryopreservation techniques have also advanced, allowing PHHs to retain their characteristics for weeks after thawing [72]. Novel models using hepatocytes isolated from humanized mouse livers have shown high infection rates, supporting the complete HBV life cycle [73]. However, PHHs cannot be sub cultured, and their long-term viability and sensitivity to HBV are limited. New methods, such as 2D and 3D cultures, are helping to overcome these challenges [74]. Spheroid culture methods, such as bioreactors or ultra-low attachment surfaces, are suitable for long-term studies of drug metabolism and virus–host interactions [75,76].

### 3.2. Human Fetal Hepatocytes

Human fetal hepatocytes, which resemble adult hepatocytes, are an attractive research system because of their functional capabilities and ability to produce vital markers (Table 1) [77]. They support HBV replication, producing viral proteins, RNA, DNA, and infectious particles [77]. However, their infection efficiency is low, and they lose sensitivity to further attacks [77]. Researchers have developed serum-free cultures that maintain hepatocyte characteristics for up to four months and co-cultured hepatocytes with nonparenchymal cells to extend liver function and HBV susceptibility for longer periods [78].

### 3.3. Huh7

The Huh7 cell line (Figure 3), derived from hepatocellular carcinoma, is used as an experimental substitute for primary hepatocytes [101]. However, Huh7 cells do not fully replicate the normal hepatocyte characteristics due to poor polarization [102]. Researchers have identified NTCP as a functional receptor for HBV and have overexpressed it in Huh7 cells to support HBV infection [67]. DMSO treatment is unnecessary for Huh7.5-NTCP cells [103], as it induces cell growth arrest [104], alters protein expression, and causes cytotoxicity [105]. Unfortunately, Huh7 cells lack support for viral uncoating and replication processes [19].

### 3.4. HepG2.2.15 Cells

The HepG2.2.15 cell line, developed by Sells et al., is a valuable system for studying HBV replication, gene expression, and antiviral drug screening (Figure 3) [60]. It was created by co-transfecting HepG2 human hepatoma cells with the recombinant vector pDoLTHBV-1, which contains two head-to-tail dimers of HBV DNA and a plasmid encoding the neomycin resistance gene [60]. The cell line can generate various HBV-specific mRNAs and produce Dane particles, which are infectious HBV virions [79]. However, it lacks the NTCP receptor, which is essential for HBV entry, making it resistant to infection. The absence of immune system components also prevents the study of host immune responses to HBV [79].

### 3.5. Vector-Based Systems

Vector-based systems play a significant role in advancing our understanding of viral replication, gene delivery, and the development of therapeutic strategies in HBV research (Figure 3). In these systems, viruses such as adenoviruses (AdVs) and adeno-associated viruses (AAVs) deliver HBV DNA to cells, particularly hepatocytes [106,107,108]. There are different types of vector-based systems, including AdVs, AAVs, recombinant HBV vectors (rHBVs), and other viral vectors, each offering distinct advantages for HBV research. AdVs are commonly used vectors to model HBV replication by delivering greater-than-genome-length HBV DNA to hepatocytes, which facilitates efficient translation, replication, and production of the virus [108,109]. AdV and rHBV systems enable control studies of HBV replication both in vitro and in vivo to assist in understanding the HBV life cycle. Additionally, HBV can be inhibited using genes delivered by AAV or rHBV vectors, which target viral proteins or stimulate the host’s immune response to clear the virus [110,111]. HBV vaccine candidates that express viral antigens and stimulate robust immune responses have been developed using AdVs, as they mimic acute infections and can be used to test vaccine effectiveness. New antiviral drugs can be tested in a controlled environment using vector systems based on HBV, such as rHBV [112]. T-cell and B-cell responses to HBV can be studied using AdV-HBV systems to mimic acute infection [113].

### 3.6. HepAD38 (EF9 and EFS19) Cells

Researchers have developed several HepG2-derived cell lines to study HBV replication and screen for potential antiviral drugs. The HepAD38 cell line, engineered by Ladner et al., contains 1.1 copies of the HBV genome and is controlled by an inducible promoter (Table 1) [79,114]. It produces significantly higher levels of HBV DNA than HepG2.2.15 cells because of a disrupted precore gene [79]. HepAD38 cells use HBeAg as a surrogate marker to estimate cccDNA levels, making them suitable for studying virus–host interactions during the early stages of HBV infection. New cell lines, HepDE19 and HepDES19, have been developed to enhance the study of HBV replication, such as the HepBHAe82 cell line for cccDNA detection and the Hep38.7-Tet cell line for higher levels of HBV replication and cccDNA [115,116].

### 3.7. Ad-HBV 1.3 System

The Ad-HBV1.3-HepG2 system, developed by He et al., is a novel method for studying HBV replication [106]. The system uses an adenoviral vector to deliver a 1.3-fold overlength HBV genome into a 293-packaging cell line and infects HepG2 cells with the recombinant virus, Ad-HBV1.3 [106]. This allows researchers to study how cells from different species can support HBV replication and the role of specific viral proteins in regulating the viral life cycle [106]. However, this system is associated with significant cytotoxicity, which may limit its use in certain research applications.

### 3.8. HepaRG Cells

The HepaRG cell line, derived from a liver tumor caused by the hepatitis C virus, is a valuable model for studying hepatic function (Figure 3) [117]. It expresses nuclear receptors and essential liver enzymes, making it more comparable to primary human hepatocytes (PHHs) [95]. HepaRG cells produce infectious HBV particles for over 100 days in a differentiated state [118]; however, HBV infection is limited. Viral replication in HepaRG cells is slow, and dimethyl sulfoxide (DMSO) is commonly used to promote differentiation [117,118]. Forskolin, an alternative to DMSO, enhances differentiation by boosting hepatic marker expression [119]. A five-chemical cocktail (5C-medium) has been employed to accelerate differentiation and maintain differentiated characteristics of iPSCs [120]. Despite the limitations of HBV infection studies, HepaRG cells are widely used in antiviral drug metabolism research because of their ability to express various CYP450 enzymes and study the entire HBV life cycle [94].

### 3.9. The 3D Culture

In vitro culturing and maintenance of hepatocytes present challenges due to their rapid loss of cuboidal morphology and liver-specific functions [121,122]. To address these issues, researchers have developed three-dimensional (3D) culture systems to promote spheroid morphology in PHHs (Figure 3) [123]. One method involves placing PHHs on a single-layer collagen matrix; however, this results in a decline in basic liver functions [124]. A novel 3D cell culture method involves the fabrication of PHH microtissues through droplet microfluidics and encapsulation of PHHs with fibroblasts [125]. This method has demonstrated sustained expression of hepatocyte genes and maintenance of functional liver-specific genes for one month or longer [126,127]. However, the quest for a robust in vitro culture system for HBV infections remains essential for studying the virus’s life cycle and developing new therapeutic strategies.

### 3.10. HBV Baculovirus System

Delaney et al. created a recombinant baculovirus/HepG2 system that enabled the expression of HBV antigens and detection of high levels of viral products (Table 1) [128,129]. The system can sustain HBV replication at elevated levels for at least 35 days, showing dose-dependent expression levels and viral infection [130]. It can quantify the impact of antiviral agents on nuclear HBV DNA and investigate the virus’s resistance to nucleoside analogues [128,129]. However, it has some limitations. A significant drawback is that traditional baculovirus vectors are not suitable for animal experiments because they are quickly inactivated by the complement system [131,132].

### 3.11. Co-Culture System

Primary human hepatocyte (PHH) cultures are useful for studying HBV infection in vitro; however, they often undergo rapid dedifferentiation, making viral infections abortive (Table 1) [87]. Zhou and colleagues developed a co-culture system that maintains hepatocyte differentiation for up to three months, preserving liver functions like bile canalicular structures [85]. Winer et al. developed a stromal cell-assisted co-culture (SACC) system that promotes advanced liver morphology and extends the functional lifespan of hepatocytes [133,134]. This system supports reproducible HBV infections and is suitable for high-throughput screening applications, allowing for the evaluation of direct-acting antivirals, host-targeting antivirals, and potentially vaccine-induced neutralizing antibodies [135] (Table 1).

### 3.12. Primary Tupaia Hepatocytes

Tree shrews, which are small mammals, are known to be susceptible to HBV infection. Primary hepatocytes from tree shrews support HBV infection, with detectable viral markers and key antigens (Table 1) [136]. The early stages of HBV infection resemble human hepatocytes, but the efficiency is low [90]. Researchers have developed a method using a recombinant adenoviral vector carrying the full HBV genome to overcome the inhibitory effect of human serum. This system allows tree shrew hepatocytes to support the complete replication cycle of HBV, including the formation of cccDNA, secretion of viral proteins, and generation of fully functional virus particles. Tree shrew hepatocytes have been pivotal in identifying NTCP as an HBV receptor [20].

Numerous in vitro cell culture systems have been developed over the past decades to investigate host viral immune responses and HBV life cycle. Nevertheless, little is known about the host response to HBV infection, and there is currently no curative treatment for HBV infections. The absence of reliable methods for culturing HBV-infected cell lines may be due to restricted host and tissue tropism [137]. Some of the drawbacks of in vitro cell culture systems are altered cell morphology, absence of an extracellular matrix, inadequate accessory cells, aberrant expression of liver enzymatic proteins, improper cell-to-cell communication, short duration of viral infection, and lack of hepatic functions after isolation and plating [62]. However, cell culture systems are the best tools for toxicity evaluation in pharmaceutical development [21,22]. Moreover, cell culture systems have contributed a great deal of knowledge and key pathways in the exploration of diseases.

## 4. In Vitro Systems Based on Induced Pluripotent Stem (iPS)

### Cell-Derived Human Hepatocytes

Human primary hepatocytes are limited in availability and tend to lose their metabolic functionality in vitro, making them less suitable for long-term studies. Hepatoma cell lines are easier to cultivate but are not susceptible to HBV infection and lack key cellular pathways. Scientists have sought alternative cell culture systems to study the HBV life cycle and its interactions with host cells [79]. One promising approach is the use of pluripotent stem cells (iPSCs), which can differentiate into various cell types and maintain genetic stability [138,139]. Researchers have also developed iPSC-derived hepatocyte lines capable of expressing NTCP, a key receptor involved in HBV entry. These hepatocyte-like cells have been used to model HBV infection and virus-induced liver dysfunction, providing a potential platform for personalized hepatitis therapies [99]. Despite challenges (the complexity of the differentiation process, strict culture requirements, and the need for advanced technical expertise), iPSC-derived systems offer promising tools for HBV research, drug development, and personalized medicine approaches.

## 5. HepG2-NTCP Cell Culture System for Studying HBV

The HepG2-NTCP cell line includes hepatoma cells engineered to express NTCP, an essential receptor for HBV and Hepatitis D viral entry. The standard HepG2 cells are not optimal for HBV infection due to the lack of the cell receptor NTCP. The HepG2-NTCP cell line has been further engineered into HepG2-NTCP sec+ cells which select for enhanced HBsAg secretion [140,141].

### 5.1. NTCP, an Effective HBV Entry Receptor

The sodium-taurocholate co-transporting polypeptide (NTCP) is encoded by the human SLC10A1 (solute carrier family 10 member 1) gene. The liver bile acid transporter (LBST) is 56 kDa in mass [142]. The HBV entry receptor NTCP was discovered in 2012, and its overexpression supports the establishment of a susceptible cell line for HBV infection. This discovery has made it possible to investigate HBV infection in greater detail and to investigate potential new treatments [20,143].

**Figure 4:**
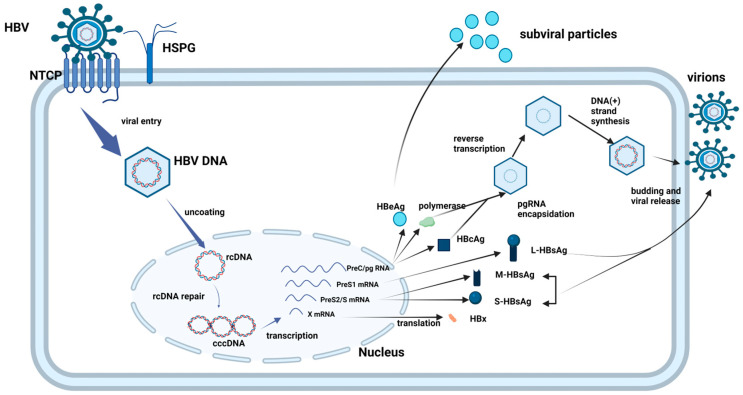
Diagrammatic representation of HBV entry into HepG2-NTCP cells via NTCP. HBV interacts with the HSPG on the cell surface and binds to the specific receptor NTCP on the HepG2-NTCP cell, and then proceeds to enter the HepG2-NTCP cell. HSPG: heparan sulphate proteoglycan; NTCP: Na+-taurocholate co-transporting polypeptide; and cccDNA: covalently closed circular DNA [79]. Created in BioRender. Mthethwa, L. (2025) https://BioRender.com/r09j085 (accessed on 18 July 2025).

NTCP is located at the hepatocyte plasma membrane on the cell’s basolateral side, where it is involved in the hepatic influx of conjugated bile salts from the portal blood circulation [142]. Primarily, NTCP binds to a complex containing two Na+ ions and bile acid for uptake; however, it can also attach to other compounds, including a range of xenobiotics, drug-conjugated bile salts, thyroid hormones, and steroid hormones. Numerous NTCP single nucleotide polymorphisms (SNPs) have been demonstrated to change the transporter’s activity; however, none of these abnormalities have been linked to serious diseases [144,145]. Knockdown studies have shown that the absence of NTCP expression in human liver cells decreases HBV infection, whereas overexpression of NTCP increases susceptibility to HBV infection [100,146,147]. The sequence of the NTCP binding site at amino acids at position 157–165 has high affinity for preS-1 of L-HBsAg, facilitating viral entry [148]. The relationship between NTCP and HBV sheds light on the early stages of viral infection and could provide a new target for antiviral therapy (Figure 4). Prior to the discovery of NTCP, heparan sulfate proteoglycan (HSPG) was thought to be the HBV adsorption mediation cell surface receptor, but this did not decode the direct link between HBV and hepatocytes [149].

### 5.2. HepG2 Cell Culture

HepG2 cells are human hepatoma cell lines. HepG2 cell lines are commonly employed in drug metabolism and hepatotoxicity research [20,150]. Moreover, HepG2 cells have recently been used in highly active antiretroviral therapy (HAART) toxicity studies [151,152]. HepG2.2.15 [60] and HepAD38 [114], two HepG2-derived cells, were used to generate cell culture-derived HBV to access the culminating phase of the viral life cycle and conduct antiviral research [80,153]. HepG2 cells’ exogenous NTCP expression facilitated the full HBV life cycle and the spread of the virus [19,57]. It has been demonstrated that HepG2-NTCP cells’ hepatocyte maintenance medium (HMM), which is produced from commercial human inducible pluripotent stem cells (iPSCs), increases HBV infection and NTCP expression [154]. HepG2-NTCP culture systems are deemed a near-perfect surrogate model for HBV studies because of their characteristics, such as good repeatability of experimental outcomes and efficient viral infection [155,156].

### 5.3. HepG2-NTCP Cell Culture

The HepG2-NTCP cell culture method offers a valuable tool for understanding different stages of HBV infection. There is even evidence suggesting that HepG2-NTCP cells are more susceptible to HBV infection then Huh7 cells expressing NTCP [19]. However, its limitations include the absence of miRNA-122 [157] and increased usage of DMSO and polyethylene glycol (PEG) [158,159,160], which are essential for sustaining viral infection in culture. König, Yang [161] demonstrated that HepG2-NTCP sec+ cells are capable of supporting the full life cycle of HBV and the long-term spread of the virus [161]. In contrast, HepG2-NTCP sec+ cells have a short-distance route for HBV to spread to nearby cells, resulting in HBV-infected cell clusters and necessitating the use of PEG and high viral titer inoculum (up to 5000 GEq/mL) to enhance infectivity. Zahoor, Kuipery [141] presented an improved in vitro infection system using the HepG2-NTCP-A3/C2 subclone in the absence of PEG [141]. HepG2-NTCP and HepG2-NTCP sec+ cell lines are promising for the exploration of HBV life cycle and pathogenesis in vitro. Moreover, the advantages of readily available, high reproducibility, and robust viral infection outweigh the limitations [39].

## 6. Phenotypic Drug Susceptibility and Resistance Testing Using Cell Culture Systems

Analyzing the functional impact of HBV mutations on antiviral efficacy requires the use of cell culture methods for phenotypic drug susceptibility and resistance testing. This approach involves introducing mutations, either naturally occurring or engineered, into HBV genomes and testing their replication capabilities in hepatoma cell lines such as HepG2 or Huh7. Cells are exposed to antiviral drugs that includes ETV or TDF to measure their inhibitory effects on viral replication [162,163]. Drug susceptibility or resistance is determined by half-maximal inhibitory concentration (IC50) values, which are derived from key outcomes such as HBV DNA levels and antigen production [162,163].

Recent studies have utilized phenotypic testing to elucidate resistance mechanisms. These studies highlight the value of phenotypic testing, prime examples being Marlet, Lier [162] demonstrating that specific polymerase mutations could significantly reduce susceptibility to ETV in the Huh7 cell line, highlighting the importance of understanding complex mutation patterns. Furthermore, Mokaya, McNaughton [164] reviewed resistance to TDF using HepG2 and Huh7 cell lines and found limited evidence for clinically significant mutations, emphasizing the need for ongoing phenotypic testing to identify rare resistance mechanisms. Moreover, Chen, Liu [163] examined the rtA181S+T184I+M204I mutation pattern in a large patient cohort using the HepG2 cell line, showing its association with multidrug resistance [163]. These findings highlight the importance of phenotypic testing in enhancing genotypic analysis and guiding treatment strategies.

## 7. HepG2 and HepG2-NTCP Cell Culture Systems in the Exploration of HBV Pathogenesis and Treatment for HBV Disease

In vitro and in vivo models have aided the investigation of the antiviral activity and potential toxicity of new substances. Knowledge of the NTCP cell surface receptor, major details of cccDNA, pgRNA degradation, and the HBx protein’s role in viral transcription has enabled investigations of multiple new therapeutic targets and a better understanding of the HBV life cycle [26,165]. However, more studies are needed to achieve the ultimate goal of a functional HBV cure. Hepatoma cell lines, especially HepG2 cells, have paved the way for researchers to study de novo HBV infection in simple and easy-to-use cell culture systems. This is made possible by the overexpression of NTCP in HepG2 cells [57]. Michailidis, Pabon [57] designed a robust cell culture system supporting the complete life cycle of HBV, including its spread, which allows for a better understanding of accurate therapeutic drug target sites.

### 7.1. HBV Entry Inhibitors

**Figure 5:**
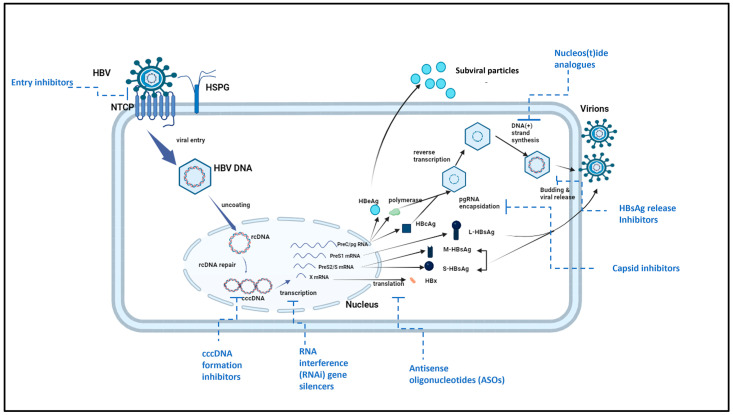
Diagrammatic representation showing where the inhibitors are acting in the replication cycle of HBV. Created in BioRender. Mthethwa, L. (2025) https://BioRender.com/r09j085 (accessed on 18 July 2025) and edited in Microsoft PowerPoint.

There are a number of studies that used HepG2 cells to investigate the inhibition of HBV entry inhibitors into hepatocytes prior to the identification of NTCP as a receptor in hepatocytes. The key mechanism for the viral entry is the interaction between the pre-S1 domain of L-HBsAg and NTCP (Figure 5) [166]. Some FDA-approved compounds that inhibit HBV entry were studied in HepG2 cell cultures. Among the most prominent and clinically advanced entry inhibitors is Myrcludex B (bulevirtide), a synthetic lipopeptide derived from the pre-S1 region of L-HBsAg. Myrcludex B binds with high affinity to NTCP, competitively blocking HBV (and hepatitis D virus) entry into hepatocytes and preventing new infections [167,168]. In addition, other compounds include the antihyperlipidemic ezetimibe [169], the immunosuppressant cyclosporin A and its derivatives [92,170], the angiotensin II receptor antagonist irbesartan [171], and the immunosuppressant rapamycin [172]. Another HepG2 cell culture study indicated that the green tea flavonoid epigallocatechin-3-gallate can effectively prevent the virus’s NTCP-mediated entrance [173]. All of the above highlight the potential of the HepG2 cell culture system in the exploration of the HBV pathogenesis and response to treatment, with Myrcludex B serving as a leading example of clinical applicability.

### 7.2. Terminal Protein Domain Inhibitors

Terminal protein domain inhibitors have been studied in HepG2 and HepG2-derived cell lines, and this is evident by the following studies: the discovery of rosmarinic acid’s ability to decrease HepG2 (and HepG2-derived) cell lines’ extracellular HBV DNA concentrations by Tsukamoto, Ikeda [174] and the identification of (*Z*)-2-(allylamino)-4-amino-*N*′-cyanothiazole-5-carboximidamide as a disruptor of ε RNA–polymerase interaction with the ability to reduced encapsidated pgRNA levels by Jo, Ryu [175]. Moreover, the studies above all demonstrated HBV DNA reduction abilities by these compounds of interest in HepG2 cell lines. There is little to no limit as to what researchers can do with a reliable HepG2-NTCP cell culture system, as they were able to demonstrate significant findings with the HepG2 cell lines above.

### 7.3. Reverse Transcriptase Inhibitors

Targeting the reverse transcriptase (RT) domain, which is the key catalytic region of the HBV DNA polymerase, has proven to be a successful strategy for NAs for inhibiting the polymerase. NAs, after being converted to triphosphate derivatives, inhibit polymerase by competing with natural deoxyribonucleotide triphosphates and incorporate into nascent DNA. NAs lack the 3′–hydroxyl group and their incorporation into nascent DNA causes DNA elongation termination (Figure 5) [176]. A prime example of such NAs was lamivudine, which was the first NA until the newer agents, entecavir and tenofovir, were introduced to overcome developing resistance.

There are several novel compounds that have been developed in recent years that have the ability to inhibit HBV DNA replication by targeting the RT domain. Most of these studies employed HepG2 cell culture systems to identify the ability of various compounds. This is evident in the work of Higashi-Kuwata, Hayashi [177], who used HepG2.2.15 cells to show that the compound (1*S*, 3*S*, 5*S*, *E*)-3-(2-Amino-6-oxo-1,6-dihydro-9H-purin-9-yl)-2-(fluoromethylene)-5-hydroxy-1-(hydroxymethyl) cyclopentane-1-carbonitrile inhibits HBV DNA production, and Zhang, Zhai [178], who demonstrated that 2′,3′-dideoxy guanosine inhibits HBV DNA replication in HepAD38 cells. Nakajima, Watashi [179] screened 1120 compounds and identified stilbene derivatives, including piceatannol as a potential anti-HBV agent, using HepG2 cells [179]. Qiu, Gong [180] employed HepG2 cells to demonstrate the ability of phenyl propionamide derivatives to inhibit HBV [180]. Parvez, Rehman [181] demonstrated that quercetin, baccatin III, psoralen, embelin, menisdaurin, and azadirachtin have the ability to inhibit hepatitis B surface antigen (HBsAg) production [181]. Ohsaki and Ueda [182] identified that suramin inhibited HBV in NTCP/G2. Further studies by Wang, Zhang [183], showed that 2-arylthio-5-iodopyrimidin analogues were good HBV polymerase inhibitors.

### 7.4. Ribonuclease H Inhibitors

Ribonuclease H (RNase H) is responsible for cleaving the RNA strand of a RNA–DNA hybrid during reverse transcription to allow complementary DNA strand synthesis. Therefore, to prevent viral replication, inhibition of RNase H is required, which prevents the cleavage of the RNA strand, leading to the accumulation of RNA–DNA duplexes inside capsids [184]. There are two classes of RNase H inhibitors (RHIs), alpha-hydroxytropolones (α-HTs) and N-hydroxyimides. Interestingly, αHTs have the ability to inhibit HIV and HBV RNase H [185,186]. Several studies have been conducted to gain a better understanding of α-HTs as RNase H inhibitors [187,188,189]. HepG2 cells and HepG2-derived cell lines have been employed to explore RHIs and their potential in the elimination of HBV. In one of the recent studies, Huber, Michailidis [190] demonstrated that analogues belonging to the class HPD inhibited HBV DNA synthesis in HepAD38 cells. Another study by Lomonosova, Zlotnick [191] examined the efficacy of RHIs (HID and α-HT) against HBV DNA synthesis in combination with 3TC and an experimental core protein allosteric modulator and found a beneficial synergistic effect in a HepG2-derived cell line [191]. In a more recent study, Chauhan, Li [192] demonstrated the suppression of cccDNA formation and inhibition of HBV during infection by RHIs in HBV-infected human hepatoma cell lines [192].

### 7.5. Inhibiting HBV Through Host-Polymerase Interactions

HBV polymerase depends on host factors such as tyrosyl-DNA phosphodiesterase (TDP2) to promote viral DNA replication, which could be targeted for the development of anti-HBV agents [193]. Inhibition of RNAi-mediated TDP2 in human cells reduces the rcDNA to cccDNA conversion rate [47]. Another host enzyme, myxovirus resistance protein 2 (MX2) inhibits the rcDNA to cccDNA conversion rate in hepatoma cells and primary hepatocytes [194]. Another additional study showed that heat shock protein 70 (HSP70) decreases capsid formation and virus particles in HepG2.2.15 cells and synergistically promotes capsid assembly with HSP90 [195]. HSPs, including HSP70 and HSP90, have been found to mediate HBV polymerase interactions [196,197,198]. Some studies have also shown that DDX3, another host factor, binds to HBV polymerase independently of pgRNA and inhibits HBV replication in hepatoma and non-hepatoma cells [199]. Although current studies demonstrating host-polymerase interactions on HepG2 cells and their derivatives are still limited, finding the essential interactions that HBV polymerase requires for replication of DNA could result in discovering new therapeutic targets that can successfully inhibit HBV infections. Therefore, more research is necessary to close the gaps and propel our knowledge of host-polymerase interactions toward the better HBV therapeutics.

### 7.6. Capsid Inhibitors

Capsid inhibitors, particularly capsid assembly modulators (CAMs), are a key class of agents being investigated for their ability to disrupt nucleocapsid formation and thereby interfere with HBV replication and cccDNA formation (Figure 5) [200,201,202]. Compounds such as GLS4 and JNJ-56136379 have demonstrated significant efficacy in HepG2.2.15 cells by reducing HBV DNA levels and preventing cccDNA formation [200,201,202]. These in vitro studies using HepG2.2.15 cells have demonstrated that CAMs can effectively reduce HBV DNA levels and prevent the establishment of cccDNA in infected hepatocytes, suggesting that CAMs could be potent antiviral agents for chronic HBV management [200,202].

### 7.7. siRNAs and Antisense Oligonucleotides (ASOs)

Small interfering RNAs (siRNAs) and ASOs represent another promising RNA interference-based therapeutic strategy targeting HBV at the transcriptional level (Figure 5). These molecules degrade HBV RNA, leading to reduced expression of viral proteins such as HBsAg [201]. HepG2-NTCP cells provide a reliable model for evaluating these RNA-targeting therapies. Evidently, the HBV RNA destabilizer AB-452 demonstrated significant antiviral activity by reducing HBV RNA, DNA, and antigens in HepG2-NTCP cells [201]. These findings suggest that further tests on siRNAs and ASOs are critical for translating these findings into clinical application.

### 7.8. cccDNA Formation Inhibitors

Inhibition of cccDNA formation is a critical strategy for achieving a functional cure for HBV. cccDNA is a persistent form of the viral genome that resides in the nucleus of infected hepatocytes (Figure 5) [203]. HepG2-NTCP cells have been instrumental in evaluating cccDNA formation inhibitors, such as disubstituted sulfonamides (CCC-0975 and CCC-0346), which impair the conversion of rcDNA to cccDNA [203]. Studies employing HepG2-NTCP cells have demonstrated that these inhibitors can effectively reduce cccDNA levels, highlighting their potential as therapeutic agents [203]. Both di-substituted sulfonamides have been tested in HepG2-derived cells and shown to significantly impair the conversion of rcDNA into cccDNA [200].

Gene editing, particularly CRISPR/Cas9, has significantly advanced the study of cccDNA formation and its inhibition in HBV research. When combined with robust and physiologically relevant cell culture systems, such as HepG2-NTCP and differentiated HepaRG cells, gene editing enables the precise manipulation of both host and viral factors that regulate cccDNA synthesis and persistence [204,205]. CRISPR/Cas9 has been used to target HBV DNA directly or to knock out host genes critical for viral replication and cccDNA maintenance, offering a unique approach for evaluating potential inhibitors of cccDNA formation [25]. These integrated systems provide a valuable platform for screening novel antiviral agents and elucidating the molecular pathways that sustain HBV infections. In hepatocyte-derived cells, Cas9 and guide RNA can be efficiently transduced using modified delivery systems, often using AAV vectors [206]. Additionally, these platforms can serve as preclinical models for developing cccDNA-targeted antiviral therapies and for evaluating gene-editing efficacy and specificity [111].

### 7.9. Genetic Targeting of Host Factors

Host-directed therapies represent another promising approach in addition to direct antiviral agents, host factors involved in the HBV life cycle are being explored as novel targets. DNA polymerase κ (POLK) has been identified as a host enzyme involved in cccDNA formation [207]. Silencing POLK expression using siRNA in HepG2-NTCP cells impairs cccDNA synthesis, providing insights into virus–host interactions and offering alternative antiviral strategies [207].

## 8. Conclusions and Future Developments

HBV infection continues to be a significant global cause of mortality. Despite the success of nucleoside analogue antivirals in HBV treatment, it remains prone to resistance and is not curative. Over the years, advancements in cell culture systems for investigating HBV pathogenesis and treatment responses have greatly benefited public health. These improved systems offer crucial insights into HBV life cycles, virus–host interactions, and therapeutic effectiveness, as highlighted in this review. The refinement of cell culture models, including innovations such as primary hepatocyte cultures, and other mentioned cell lines, has enabled more accurate study outcomes. Consequently, these models have been essential in enhancing preclinical research, supporting the development of more effective treatments and vaccines against HBV. In vitro cell culture systems have potential for exploring molecular mechanisms of the complex HBV genetic diversity and its diverse clinical presentations. Furthermore, the HepG2 cell line and its derivatives have paved the way for identifying multiple drug targets. This is evident by the studies presented above. However, there is still a long way to go in research to achieve the goal of eliminating HBV as a health threat. The development of therapeutic targets for antiviral agents relies on in vitro cell culture systems as the first stage in drug development. The discovery of NTCP as a receptor for HBV [19,20] allowed researchers to develop the HepG2-NTCP cell culture system, supporting the study of the complete life cycle and spread [57]. This opened an array of opportunities to study HBV pathogenesis and its potential cure using a relatively simple and reliable system.

## Figures and Tables

**Table 1:** Summary of HBV in vitro hepatocyte culture models [79], highlighting advantages and limitations.

Cell Line	Advantages	Shortcomings	HBV Infection Rate and Application of the Models
HepG2.2.15 cells	cccDNA accumulation. Continuous HBV gene expression and replication.	Reduced viral replication. Unstable antigen expression. Virion production from integrated DNA.	Antiviral compound screening and assessment, etc. [80].
HepAD38 (EF9 and EFS19) cells	These cells differentiate rapidly and produce significant quantities of viral particles. Importantly, they also allow for the accumulation of covalently closed circular DNA (cccDNA), a crucial aspect of the HBV life cycle.	Virions are created from integrated DNA and represent an incomplete viral life cycle.	The model is useful for studying HBV infection, cccDNA persistence, and exploring antiviral treatment strategies in a controlled lab setting. A possible source of virions produced from tissue culture [81].
Ad-HBV1.3-systems	No species barrier. Effective HBV expression. Controllable HBV expression and mutation. Direct measurement of the efficacy of transfection and infection (integrated green fluorescent protein gene).	Lacking the normal infection stage of HBV.	Used to create acute hepatitis B infection models in animals [82].
HBV baculovirus system	Easy detection of riboprotein-bound HBV DNA. High HBV replication level. Formation of infectious viruses and a detectable intracellular cccDNA pool.	Nonreceptor-mediated entry. Gene transfer is restricted to certain species. Missing HBV natural infection stage.	Quantify the effect of antiviral agents on nuclear HBV DNA. Used for studying the resistance of HBV to nucleoside analogues [83].
Primary human hepatocytes (PHH)	Supports the full life cycle of HBV infections: Capable of replicating every stage of infection, from viral entry and replication to release. Includes a variety of liver-specific host factors: These hepatocyte-specific components ensure that the model closely mimics the natural infection process in human liver cells. Has a fully functional innate immune system: This feature allows the system to simulate the body’s initial immune response to the viral infections, facilitating the study of immune evasion and antiviral defenses.	Scarcity of high-quality donors and limited cell lifespan. Varying degrees of susceptibility to HBV infections. Loss of functional characteristics following plating. Challenges in maintaining appropriate culture conditions.	Assessment of drug candidates’ toxicity, drug–drug interactions, drug transporter activity, and metabolism in vitro.
Human fetal hepatocytes	Phenotypically and biologically functionally close to primary adult human hepatocytes.	Low infection efficiency. Short infection time. Limited availability. Large donor–donor variations.	HBV infection rate 12–90% [77,84]. Co-culturing with hepatic non-parenchymal cells and subsequent addition of 2% DMSO leads to the formation of hepatocyte islands with prolonged phenotypic maintenance [85]. The early events in viral entry into cells as well as viral replication [86].
Adult human hepatocytes	The gold standard host cell to HBV infection experiments. Closest to the physiological characteristics of hepatocytes in vivo. Close to the natural process of infection.	Limited life cycle. Unpassable culture. Phenotypically unstable in vitro. Rapidly lose permissiveness for HBV Infection. Large donor–donor variations.	HBV infection rate 20–100% [71,87]. Used for studying the process of HBV infection [62,87]. Used for studying apoptosis [71]. Preparation of 3D primary hepatocyte culture system for analyses of liver diseases, drug metabolism, and toxicity [75,88].
Co-culture system	Test the utility of various direct-acting antivirals (DAAs) and putative host-targeting antivirals (HTAs). Assessing preclinically the efficacy of other entry inhibitors and possibly (vaccine-induced) neutralizing antibodies.	Wide variability between donors in terms of HBV permissiveness.	Inflammation and drug-induced hepatotoxicity [89].
Primary Tupaia hepatocytes	The only species susceptible to HBV infection besides humans and chimpanzees.	Expensive.	HBV infection rate > 70% [90]. Used for in vitro as well as in vivo infection experiments [91]. HBV-specific receptor identification [92].
HepaRG cells	Preserve the specific functional properties of hepatocytes. Support the complete HBV life cycle. Produce HBV cccDNA. Involved in liver functions. Produces transcripts for a variety of nuclear receptors.	Strict culture conditions. Exhibits low infection efficiency. Requires cellular differentiation. Limited ability for cell-to-cell transmission.	HBV infection rate < 30% [92,93]. HBV molecular mechanism and screening, evaluation of anti-HBV drugs, cccDNA spread, etc. [94]. Drug metabolism and toxicity [95,96].
In vitro systems based on induced pluripotent stem (iPS) cell-derived human hepatocytes	Exhibit characteristics closely resembling those of healthy liver cells capable of supporting the entire viral replication cycle. Possess a fully functional immune response system.	Complex procedure.	HBV infection rates can reach as high as 25% [97]. Screening for drug-induced hepatotoxicity [98]. The HBV virus’s life cycle and the damage it causes to the liver [99].
NTCP overexpressing hepatoma cell lines	Supporting the full life cycle of a virus. Flexibility and ease of use.	The cells exhibit low sensitivity to infection by HBV derived from serum. Achieving infection requires a very high multiplicity of infection (MOI). After infection occurs, there is minimal viral spread to other cells.	HBV infection rates can reach as high as 50% [100]. Screen antiviral drugs on a large scale, with a focus on targeting the NTCP receptor, which is essential for HBV entry into liver cells [81].
Huh7-NTCP	Enhanced infection efficiency.	Only partially replicate the behavior of normal hepatocytes due to inadequate polarization. Lacks detectable levels of the receptor.	Useful for studies of the HBV virus.
HepG2-NTCP	Easily accessible. Consistent reproducibility. Strong viral infection.	Incomplete mimicry of normal hepatocyte function. Reduced viral replication and infection efficiency. Necessitates additional use of PEG and DMSO for optimal results.	HBV studies. In vitro evaluation of metabolism.
HepG2-NTCP sec+	Complete HBV life cycle support. Sustained viral propagation.	Requires a high viral titer for effective inoculation. Needs PEG to enhance viral infectivity.	HBV studies. In vitro evaluation of metabolism.
The 3D culture	Preserves cell morphology. No PEG or DMSO requirement.	Does not fully replicate the natural hepatic environment or maintain liver-specific functions.	HBV studies.

## Abbreviations

ARV	antiretroviral
AASLD	American Association for the Study of Liver Diseases
ASOs	antisense oligonucleotides
ART	antiretroviral therapy
ALT	alanine aminotransferase
α-HTs	alpha-hydroxytropolones
CAMs	capsid assembly modulators
cccDNA	covalently closed circular deoxyribonucleic acid
CYP450	cytochrome P450 enzymes
CMV-IE	cytomegalovirus immediate-early
DAAs	direct-acting antivirals
DNA	deoxyribonucleic acid
DMSO	dimethyl sulfoxide
FTC	emtricitabine
FDA	Food and Drug Administration
GFP	green fluorescent protein
IC50	half-maximal inhibitory concentration
HBx	HBV X protein
HBV	Hepatitis B virus
HCV	hepatitis C virus
HBeAg	hepatitis B e antigen
HBcAg	hepatitis B core antigen
HBsAg	hepatitis B surface antigen
HSPGs	heparan sulphate proteoglycans
HLCs	hepatocyte-like cells
HSPG	heparan sulphate proteoglycan
HCC	hepatocellular carcinoma
HMM	hepatocyte maintenance medium
HepG2 cells	human hepatoblastoma cell line
HepG2-NTCP cell	HepG2-sodium taurocholate co-transporting polypeptide
HepaRG cells	human hepatic bipotent progenitor cell line
Huh7	human liver carcinoma cell line
Huh7-NTCP	Huh7-sodium taurocholate co-transporting polypeptide
HepAD38 (EF9,EFS19) cells	liver-derived cell lines
HepG2.2.15 cells	human hepatoblastoma cell line
HSP90	heat shock protein 90
HSP70	heat shock protein 70
HAART	highly active antiretroviral therapy
HTS	high-throughput screening
HTAs	host-targeting antivirals
HIV	human immunodeficiency virus
iPSCs	inducible pluripotent stem cells
3TC	lamivudine
miRNA-122	microRNA-122
MOI	multiplicity of infection
MX2	myxovirus resistance protein 2
NAs	nucleos(t)ide analogues
ORF	open reading frame
PHH	primary human hepatocyte
PEG	poly-ethylene glycol
pgRNA	pregenomic RNA
POLK	DNA polymerase κ
RT	reverse transcriptase
RNase H	ribonuclease H
RHIs	RNase H inhibitors
rcDNA	relaxed circular deoxyribonucleic acid
siRNAs	small interfering RNAs
SNPs	single nucleotide polymorphisms
NTCP	sodium taurocholate co-transporting polypeptide
TDF	tenofovir disoproxil fumarate
TAF	tenofovir alafenamide
EASL	European Association for the Study of the Liver
APASL	Asian Pacific Association for the Study of the Liver
TP	terminal protein
2D	two-dimensional
3D	three-dimensional
TDP2	tyrosyl-DNA-phosphodiesterase 2
CDC	U.S. Centers for Disease Control and Prevention
